# Multifunctional Platform Based on Electroactive Polymers and Silica Nanoparticles for Tissue Engineering Applications

**DOI:** 10.3390/nano8110933

**Published:** 2018-11-09

**Authors:** Sylvie Ribeiro, Tânia Ribeiro, Clarisse Ribeiro, Daniela M. Correia, José P. Sequeira Farinha, Andreia Castro Gomes, Carlos Baleizão, Senentxu Lanceros-Méndez

**Affiliations:** 1 Centro/Departamento de Física, Universidade do Minho, 4710-057 Braga, Portugal; sribeiro@fisica.uminho.pt; 2 Centre of Molecular and Environmental Biology (CBMA), Universidade do Minho, Campus de Gualtar, 4710-057 Braga, Portugal; agomes@bio.uminho.pt; 3 Centro de Química-Física Molecular and Institute of Nanosciences and Nanotechnology, Instituto Superior Técnico, Universidade de Lisboa, 1049-001 Lisboa, Portugal; tania.ribeiro@tecnico.ulisboa.pt (T.R.); farinha@tecnico.ulisboa.pt (J.P.S.F.); carlos.baleizao@tecnico.ulisboa.pt (C.B.); 4 Centro de Química Estrutural, Instituto Superior Técnico, Universidade de Lisboa, 1049-001 Lisboa, Portugal; 5 CEB—Centre of Biological Engineering, Universidade do Minho, Campus de Gualtar, 4710 057 Braga, Portugal; 6 Chemical Department and CQ-VR, Universidade de Trás-os-Montes e Alto Douro, 5001-801 Vila Real, Portugal; d.correia@fisica.uminho.pt; 7 BCMaterials, Basque Centre for Materials, Applications and Nanostructures, UPV/EHU Science Park, 48940 Leioa, Spain; senentxu.lanceros@bcmaterials.net; 8 IKERBASQUE, Basque Foundation for Science, 48013 Bilbao, Spain

**Keywords:** nanostructures, polymer matrix composites (PMCs), mechanical properties, thermal properties

## Abstract

Poly(vinylidene fluoride) nanocomposites processed with different morphologies, such as porous and non-porous films and fibres, have been prepared with silica nanoparticles (SiNPs) of varying diameter (17, 100, 160 and 300 nm), which in turn have encapsulated perylenediimide (PDI), a fluorescent molecule. The structural, morphological, optical, thermal, and mechanical properties of the nanocomposites, with SiNP filler concentration up to 16 wt %, were evaluated. Furthermore, cytotoxicity and cell proliferation studies were performed. All SiNPs are negatively charged independently of the pH and more stable from pH 5 upwards. The introduction of SiNPs within the polymer matrix increases the contact angle independently of the nanoparticle diameter. Moreover, the smallest ones (17 nm) also improve the PVDF Young’s modulus. The filler diameter, physico-chemical, thermal and mechanical properties of the polymer matrix were not significantly affected. Finally, the SiNPs’ inclusion does not induce cytotoxicity in murine myoblasts (C2C12) after 72 h of contact and proliferation studies reveal that the prepared composites represent a suitable platform for tissue engineering applications, as they allow us to combine the biocompatibility and piezoelectricity of the polymer with the possible functionalization and drug encapsulation and release of the SiNP.

## 1. Introduction

The development of advanced multifunctional materials is essential for the development of society [1]. Nanocomposites are among the most important materials for an increasing number of applications due to the possibility of designing materials with tailored properties meeting specific application demands in areas ranging from the automotive industry [2,3] to food packaging [4,5] and tissue engineering [6,7], among others. The introduction of inorganic nanomaterials into polymers allows for the combination of the rigidity and high thermal stability of the inorganic material with the ductibility, flexibility and processability of the organic polymers [8], as well as the introduction/tuning of further functionalities such as magnetic [9], mechanical [10] or electrical properties [11,12]. Typical nanomaterials include nanoparticles, nanotubes, nanofibres, fullerenes and nanowires [13]. Among nanomaterials, silica is widely present in the environment and has several key features [14].

Properties of silica nanoparticles (SiNPs), such as high mechanical strength, permeability, thermal and chemical stability, relatively low refractive index, high surface area, and the fact of being used for coatings of other particles, such as magnetic and quantum dots [15,16,17], make these nanoparticles highly interesting for various applications [18]. Furthermore, their biocompatibility and the different possibilities of functionalizing them are the basis of their potential for biomedicine and tissue engineering applications [19]. Silica nanostructures have been extensively used as supports or carriers in drug delivery [20,21], nanomedicine [22,23] and bioanalysis [24]. Their characteristics can be tuned during synthesis to obtain a wide range of particle diameters ranging from 20 to 500 nm, different pore sizes and the incorporation of molecules such as drugs or fluorophores [24], as well as magnetic nanostructures [25]. Mesoporous silica nanoparticles (MSNPs) [20,26] have attracted particular attention for their functionalization versatility. Silica-based mesoporous nanoparticles, due to the strong Si‒O bond compared to niosomes, liposomes and dendrimers, are more resistant to degradation and mechanical stress, obviating the need for any external stabilization of the MSNPs [27,28].

With respect to tissue engineering, different tissues require different microenvironments for suitable regeneration [29]. Thus, muscle tissue has electromechanical responses and needs electrical stimulation to support ionic exchange, mainly sodium by calcium ion [30]. In this context, electroactive polymers such as magnetoelectric [31,32], piezoelectric and conductive polymers [33], among others [34], show strong potential for tissue engineering applications. Among the different electroactive polymers, piezoelectric polymers have already shown their suitability for tissue engineering [6,29] due to their capacity to vary surface charge when a mechanical load is applied or vice versa. These materials can play a significant role because electric stimulation can be found in many living tissues of the human body, namely nerves [35] and bones [36,37], and it can provide the electromechanical solicitations for muscle tissue [38]. Poly(vinylidene fluoride) (PVDF) is the biocompatible piezoelectric polymer with the highest piezoelectric response. It can crystallize in four differentiated crystalline phases, α, β, γ, δ, with the β-phase being the one with the highest piezoelectric coefficient. Furthermore, it can be processed in different morphologies, including fibres, spheres, membranes and 3D scaffolds [29,39], providing a suitable platform for tissue engineering.

In order to further exploit the applicability of PVDF in regenerative medicine, polymer nanocomposites based on PVDF using silica nanoparticles with different diameters were prepared, improving the electroactive characteristics of PVDF with the aforementioned characteristics of MSNPs for biomedical applications. Together with the physico-chemical characteristics of the developed composites, their biocompatibility was evaluated in murine myoblast cells.

## 2. Materials and Methods

### 2.1. Materials

PVDF (Solef 1010) was purchased from Solvay, *N*,*N*-dimethylformamide (DMF) from Merck. Absolute ethanol (EtOH, Panreac, Barcelona, Spain, 99.5%), ammonium hydroxide solution (NH_4_OH, 28% in water, Fluka, Carnaxide, Portugal) and tetraethyl orthosilicate (TEOS, Sigma-Aldrich, Sintra, Portugal, 99%) were used as received. Deionized water from a Millipore (Oeiras, Portugal) system Milli-Q ≥ 18 MΩ cm was used in the synthesis of the silica nanoparticles. Perylenediimide derivative (PDI) was synthesized according to the literature [40].

### 2.2. Silica Nanoparticles

#### 2.2.1. Preparation of the Silica Nanoparticles

Fluorescent silica nanoparticles, doped with PDI were prepared by a modified Stöber method [41,42]. Water, absolute ethanol, and PDI (previously dispersed in ethanol, 1 × 10^−6^ M) were mixed and after 30 min the ammonia solution was added to the mixture, followed by TEOS. The reaction was kept under stirring at constant temperature for 24 h. After that time, the nanoparticles were recovered and washed with ethanol (three cycles of centrifugation). The nanoparticles were redispersed in ethanol and dried at 50 °C in a ventilated oven. The experimental details are provided in Table 1. 

#### 2.2.2. Characterization of the SiNPs

Transmission electron microscopy: Transmission electron microscopy (TEM) images were obtained on a Hitachi (Krefeld, Germany) transmission electron microscope (model H-8100 with a LaB6 filament) with an acceleration voltage of 200 kV. One drop of the dispersion of particles in ethanol was placed on a carbon grid and dried in air before observation. The images were processed with the Fiji software (Madison, WI, USA).

Zeta potential: The surface charge of the nanoparticles was estimated with the use of zeta potential (Zetasizer NANO ZS-ZEN3600, Malvern). The zeta potential of the fluorescent SiNPs with different diameters were evaluated at different pH values (3, 5, 7, 11, 13). To adjust the pH, it was used a solutions of HCl (1M) and NaOH (1M). The average value and standard deviation for each sample were obtained from six measurements.

### 2.3. Nanocomposite Samples

#### 2.3.1. Preparation of the SiNPs/PVDF Nanocomposites

SiNPs/PVDF nanocomposites with 16 wt % of SiNPs were prepared by dispersing the respective mass of SiNPs in the DMF solvent within an ultrasound bath for 4 h at room temperature. The filler concentration was selected based in [43], as it shows a suitable filler content without compromising the mechanical characteristics of the polymer matrix and allowing a suitable dispersion of the filler. After we obtained a good dispersion of the nanoparticles, PVDF was added ay a concentration of 15% (*w*/*w*) and the solution was magnetically stirred at room temperature until the complete dissolution of the polymer. The materials were then prepared by different production methods [39].

First, SiNPs/PVDF samples (porous and non-porous films) were prepared by solution casting on a clean glass substrate and, in some cases, melted at different temperatures for different times (Table 2). The different preparation conditions allowed us to tailor the porosity and to study the possibility of the nucleation of the electroactive β-phase of the polymer by the fillers [44]. The thickness of the films ranges from 30 to 50 µm.

For SiNPs/PVDF electrospun fibre mats, the solution was placed in a plastic syringe (10 mL) fitted with a steel needle with inner diameter of 0.5 mm. After an optimization procedure, electrospinning was conducted with a high-voltage power supply from Glasman (model PS/FC30P04, Radeberg, Germany) at 14 kV with a feed rate of 0.5 mL·h^−1^ (with a syringe pump from Syringepump, Porto, Portugal). The electrospun fibres were collected in an aluminium plate (placed 20 cm from the needle) and in a rotating drum (1500 rpm) to obtain random and oriented nanofibres, respectively.

Table 2 summarizes the main characteristics of the samples and the corresponding denomination that refers the type of sample and processing temperature, the nanoparticle diameter and the composite morphology. For example, F90-17NP is a film (F) obtained at 90 °C (90) with nanoparticles with a diameter of 17 nm (17), which is non-porous (NP).

#### 2.3.2. Characterization of the Nanocomposite Samples

Scanning electron microscopy: A desktop scanning electron microscope (SEM) (Phenom ProX, Eindhoven, The Netherlands) was used to observe the morphology and microstructure of the PVDF and SiNPs/PVDF nanocomposites. This technique was also used to observe the cell morphology seeded on the different fibrous samples. All the samples were added to the aluminium pin stubs with electrically conductive carbon adhesive tape (PELCO TabsTM, Redding, CA, USA). The aluminium pin stub was then placed on a phenom Charge Reduction sample Holder. All results were acquired using the ProSuite software (Waarschoot, Belgium). The images were obtained with an acceleration voltage of 10 kV. 

Laser scanning confocal fluorescence microscopy: Laser scanning confocal fluorescence microscopy (LSCFM) images were obtained with a Leica TCS SP5 laser scanning microscope (Leica Microsystems CMS GmbH, Mannheim, Germany) using an inverted microscope (DMI6000), a HCX PL APO CS 10× dry immersion objective (10× magnification and 0.4 numerical aperture) and a HC PL FLUOTAR 50× dry immersion objective (50× magnification and 0.8 numerical aperture). Imaging used the 488 nm line of an argon ion laser.

Contact angle measurements: Water contact angle (CA) measurements (sessile drop in dynamic mode) were performed at room temperature in a Data Physics OCA20 (Data Physics, Filderstadt, Germany) setup using ultrapure water as the test liquid. The samples wettability was determined by using water drops (3 μL) placed onto the surface of the samples. Each sample was measured at six different locations and the mean contact angle and standard deviation were calculated.

Fourier transform infrared spectroscopy: Fourier transform infrared spectroscopy (FTIR) measurements in attenuated total reflectance (ATR) were performed at room temperature, using a Nicolet Nexus 670 FTIR-spectrophotometer (ThermoFisher Scientific, Porto Salvo, Portugal) with Smart Orbit Accessory equipment (ThermoFisher Scientific, Porto Salvo, Portugal). The analysis was performed from 4000 to 600 cm^−1^, after 64 scans with a resolution of 4 cm^−1^. The spectra of each sample was used to determine the relative content of the electroactive β-phase in the composite samples, by using the method presented in [44]. In short, the ^®^-phase content (F^®^) was calculated by Equation (1):

(1) $$F_{\beta }=\frac{A_{\beta }}{\left(\frac{K_{\beta }}{K_{\alpha }}\right)\times A_{\alpha }+A_{\beta }},$$

where $A_{\beta }$ are the absorbance at 840 cm^−1^ and *K_β_* = 7.7 × 10^4^ cm^2^·mol^−1^ is the absorption coefficients and correspond to the β phase. A_α_ is the absorbance at 760 cm^−1^ and *K_α_* = 6.1 × 10^4^ cm^2^·mol^−1^ is the absorption coefficient, and correspond to the α phase.

Thermal properties: Differential scanning calorimetry (DSC) was carried out with a DSC 6000 Perkin Elmer (Mettler Toledo, Columbus, OH, USA) instrument. The samples were heated from 30 to 200 °C at a rate of 10 °C·min^−1^ under a flowing nitrogen atmosphere. Samples were cut from the middle region of the samples and placed in aluminium pans.

From the melting in the DSC thermograms, the degree crystallinity (*X_c_*) of the samples was calculated by the following equation [44]:

(2) $$X_{c}=\frac{\Delta H_{f}}{x\Delta H_{\alpha }+y\Delta H_{\beta }},$$

where $\Delta H_{f}$ is the melting enthalpy of the sample, *x* and *y* represent the *α* and *β* phase contents present in the sample, respectively, and $\Delta H_{\alpha }$ and $\Delta H_{\beta }$ are the melting enthalpies for a 100% α-PVDF (93.04 J·g^−1^) and β-PVDF (104.4 J·g^−1^) crystalline samples respectively.

Mechanical characterization: Mechanical measurements were performed with a universal testing machine (Shimadzu model AG-IS, Kyoto, Japan) at room temperature, in tensile mode at a test velocity of 1 mm·min^−1^, with a load cell of 50 N. The tests were performed on rectangular samples (30 × 10 mm) with a thickness between 30 and 50 μm (Fischer Dualscope 603-478, digital micrometer, Windsor, CT, USA). The mechanical parameters were calculated from the average of triplicate measurements. Hook’s law was used to obtain the effective Young’s modulus (E) of PVDF and SiNPs/PVDF nanocomposite samples in the linear zone of elasticity between 0 and 1% strain.

### 2.4. Cell Culture Experiments

#### 2.4.1. Sample Sterilization

The samples were sterilized by multiple immersions into 70% ethanol for 30 min each and to remove any residual solvent, they were washed five times in a phosphate buffered saline (PBS) 1× solution for 5 min each. Each side of the samples was then exposed to ultraviolet (UV) light for 1 h.

#### 2.4.2. Cell Culture

Murine myoblasts (C2C12 cell line) were cultivated in Dulbecco’s Modified Eagle’s Medium (DMEM, Gibco, Porto Salvo, Portugal) with 4.5 g·L^−1^ containing 10% of Foetal Bovine Serum (FBS, Biochrom, Cambridge, UK) and 1% of Penicillin/Streptomycin (P/S, Biochrom). The cells were grown in a 75 cm^2^ cell-culture flask at 37 °C in a humidified air containing 5% CO_2_ atmosphere. Every two days, the culture medium was changed. The cells were trypsinized with 0.05% trypsin-EDTA when they reached 60–70% confluence. For the cytotoxicity assays, SiNPs/PVDF nanocomposites with different morphologies were cut according to the ISO_10993-12. The extraction ratio (surface area or mass/volume) was 6 cm^2^.mL^−1^. To analyse cell morphology and viability, the materials were cut into 6 mm diameter. PVDF films without nanoparticles were used as the control.

#### 2.4.3. Cytotoxicity Assay by the Indirect Contact

C2C12 cells were seeded at the density of 2 × 10^4^ cells·mL^−1^ in 96-well tissue culture polystyrene plates. Cells were allowed to attach for 24 h, after which the culture medium was removed and the conditioned medium (the medium that was in contact with the samples) was added to the wells (100 µL). Afterwards, the cells were incubated for 24 or 72 h, and the number of viable cells was quantified by (3-(4,5-Dimethylthiazol-2-yl)-2,5-diphenyltetrazolium bromide) (MTT) assay. The cells received MTT solution (5 mg·mL^−1^ in PBS dissolved in DMEM in proportion of 10%) and were incubated in the dark at 37 °C for 2 h. The medium was then removed and 100 µL of DMSO/well were added to dissolve the precipitated formazan. The quantification was determined by measuring the absorbance at 570 nm using a microplate reader. All quantitative results were obtained from four replicate samples and controls and were analysed as the average of viability ± standard deviation (SD).

#### 2.4.4. Direct Contact and Proliferation

Since MTT interferes with the materials, we chose the MTS as having the same theoretical basis but a soluble reaction product. C2C12 cells (4000) were seeded on each sample. After 24 h and 72 h, the viable cell number was determined using the (3-(4,5-dimethylthiazol-2-yl)-5-(3-carboxymethoxyphenyl)-2-(4-sulfophenyl)-2H-tetrazolium) (MTS) assay. At the desired time points, the MTS reagent was added into each well in a 1:5 proportion of DMEM medium, and incubated at 37 °C for 2 h. The absorbance was detected at 490 nm with a microplate reader. Experimental data were obtained from four replicates.

#### 2.4.5. Immunofluorescence Staining

Using the same time points as in the proliferation assays, the nanocomposite samples were subjected to immunofluorescence staining to analyse the cytoskeleton morphology of the cells, also verifying the cell viability and adhesion. At each time point, the medium of each well was removed, the samples were washed with PBS and the cells fixed with 4% formaldehyde for 10 min at 37 °C in a 5% CO_2_ incubator. After fixation, the samples were washed with PBS 1× (three times) and incubated for 45 min at room temperature in 0.1 µg mL^−1^ of green phalloidin (Sigma-Aldrich, Sintra, Portugal). Then, the samples were incubated for 5 min with 1 µg mL^−1^ of 4,6-diamidino-2-phenylindole (DAPI, Sigma). Afterwards, the samples were washed again with PBS 1× (three times) and one time with distillate water. Finally, the samples were visualized with fluorescence microscopy (Olympus BX51 Microscope, Lisboa, Portugal).

## 3. Results and Discussion

### 3.1. Silica Nanoparticles

#### 3.1.1. Morphology and Size of the Nanoparticles

**Figure 1:**
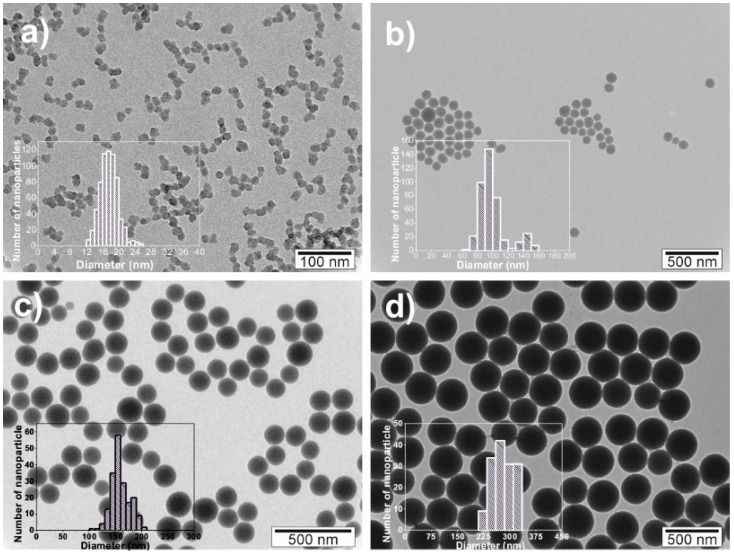
TEM images of SiNPs-PDI with different particle size: (**a**) 17 ± 2 nm, (**b**) 100 ± 1 m, (**c**) 160 ± 1 m and (**d**) 300 ± 3 m.

The morphology and the size of the SiNPs were analysed from TEM images (Figure 1). The spherical nanoparticles prepared by the Stober method [45] were prepared in four different diameters: 17 ± 2, 100 ± 18, 160 ± 17 and 300 ± 37 nm. The corresponding histograms are presented as insets in Figure 1.

#### 3.1.2. Surface Charge of the Nanoparticles

**Figure 2:**
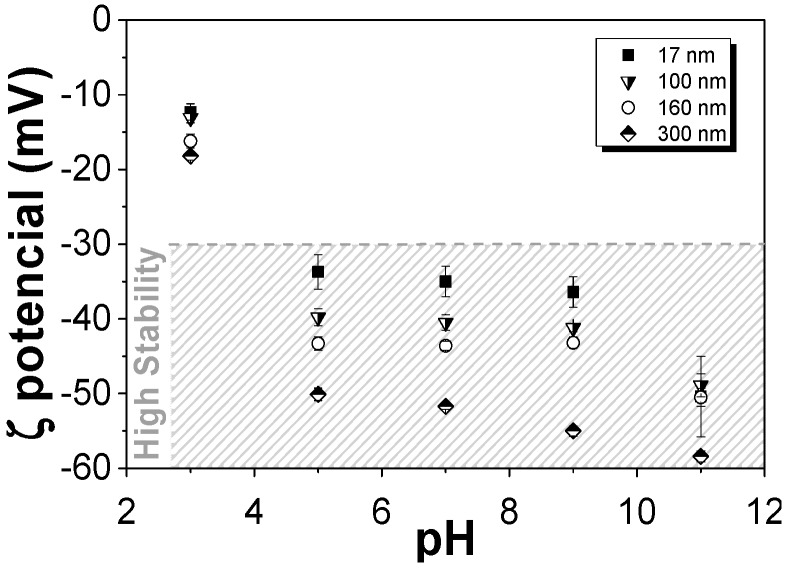
Zeta potential of the different SiNPs nanoparticles at different pH.

Figure 2 shows the zeta potential of aqueous dispersions of the different SiNPs at different pH to analyse the periphery charge of the particles.

The particles are considered more stable with a zeta potential above +30 mV or below −30 mV. This fact is due to the electrostatic repulsions between the nanoparticles that prevent their aggregation. Figure 2 shows that all nanoparticles are more stable at pH ≥ 5, independently of their average diameter. On the other hand, nanoparticles with higher average diameters are more stable. The isoelectric point of SiNPs is close to pH 2 so, from this pH upwards, the silica nanoparticles are negatively charged in acidic, neutral and basic environments, which can be taken advantage of as it has been demonstrated that the interactions between negatively charged nanoparticle surfaces and the positive charge density of the CH_2_ groups of the PVDF polymer can promote the nucleation of the electroactive β-phase of the polymer [46].

### 3.2. SiNPs/PVDF Nanocomposite Samples

#### 3.2.1. Morphology of the Nanocomposites

**Figure 3:**
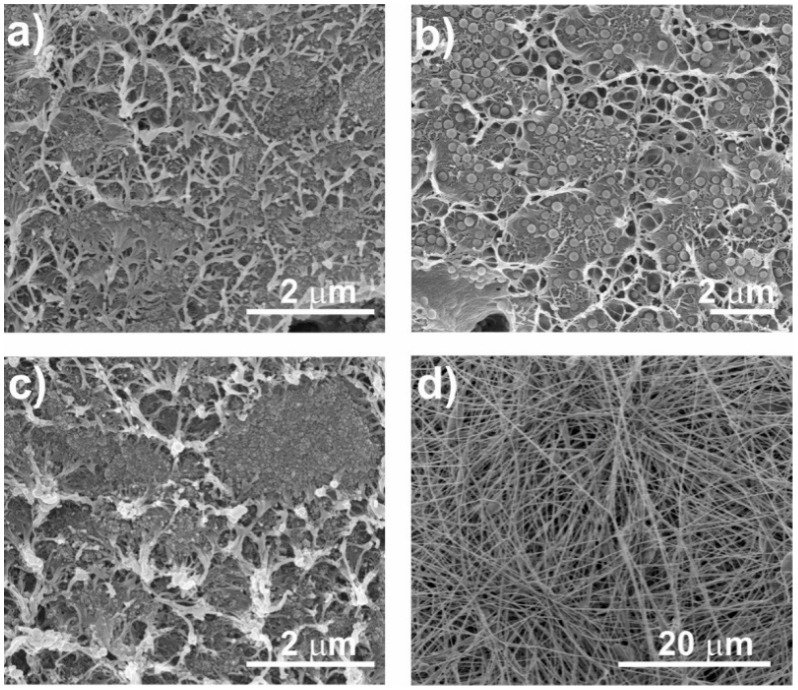
Cross section SEM micrographs of SiNPs/PVDF nanocomposite samples with nanoparticles of different diameters and different processing conditions: (**a**) F90-17NP, (**b**) F90-300NP, (**c**) F210-17NP, (**d**) R-17P.

The morphology of the nanocomposites was assessed by SEM. Figure 3 shows the different morphologies obtained after the different processing methods as well as the variations due to the introduction of fillers with different diameters. Figure 3 shows the cross section (Figure 3a–c) of the nanocomposites and electrospun fibres samples (Figure 3d) with 16 wt % of SiNPs. Figure 3a,b present the differences between the samples obtained at 90 °C with SiNPs of different diameters, showing that the higher diameter particles are well-dispersed in the PVDF polymer matrix, in contrast to the SiNPs with lower diameter that present particle agglomerates. Furthermore, a small porosity is observed (Figure 3a), which is in agreement with the literature [47].

It is important to note that the nanoparticles act as nucleation agents for crystallization in PVDF composites [48], which can be verified with the results obtained, indicating a good interfacial interaction between the PVDF chains and silica nanoparticles.

Figure 3a,c shows the differences in composite morphology due to the crystallization process. The samples obtained at 90 °C (Figure 3a,b) present a slightly more porous morphology than the ones obtained at 210 °C (Figure 3c).

Once the SiNPs of 17 nm do not show a suitable dispersion in the films, electrospinning was used in order to produce fibres with well-dispersed particles. Relative to the fibres (Figure 3d), smooth randomly oriented fibres with encapsulated particles are observed, with no particles at the surface.

**Figure 4:**
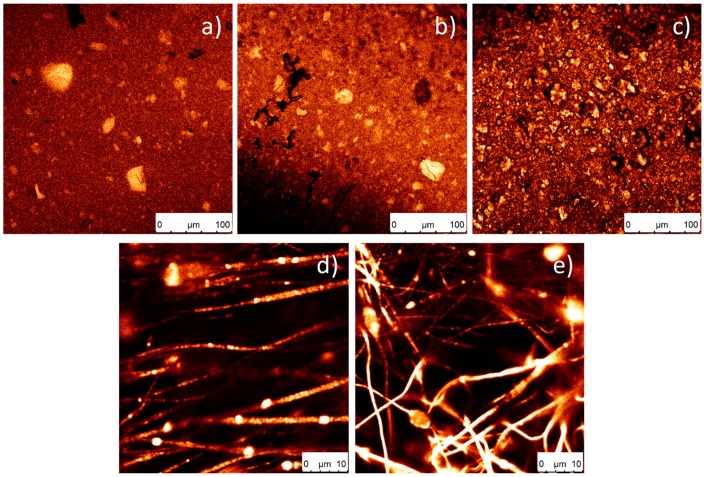
Representative confocal images of SiNPs/PVDF nanocomposites with different morphologies: (**a**) F210-17NP, (**b**) F90-17NP, (**c**) Ftrt-17P, (**d**) O-17P and (**e**) R-17P.

This result is confirmed by the confocal images represented in Figure 4. It was observed that the introduction of the particles increases the fibre diameter (243 ± 89 nm to 339 ± 92 nm). Oriented fibres with SiNPs were also produced (data not shown), verifying the particles’ encapsulation within the fibres and a fibre diameter of 683 ± 140 nm. The increase of fibre diameter with the incorporation of the SiNPs is attributed to the higher viscosity of the solution, with also hinders fibre stretching by the applied field. The higher diameter of the oriented fibres relative to the randomly oriented fibres is attributed to the merging of aligned fibrils that crystallize simultaneously [49].

#### 3.2.2. Confocal Fluorescence Microscopy of the Nanocomposites

The incorporation of PDI in the silica nanoparticles can increase their application range, in particular, for biomedical applications, as it allows their tracking and localization [42,50]. In Figure 4, the green identifies the fluorescence of the nanoparticles; a higher colour intensity indicates a higher number of nanoparticles present. Figure 4a–c shows that, as the processing temperature decreases, a larger aggregation of nanoparticles is observed. In Figure 4a, where the temperature is higher, more homogeneous samples were obtained.

Relative to the oriented and random fibres (Figure 4d,e, respectively), it is observed that the nanoparticles are present and included within the fibres.

#### 3.2.3. Wettability of the Nanocomposites

**Figure 5:**
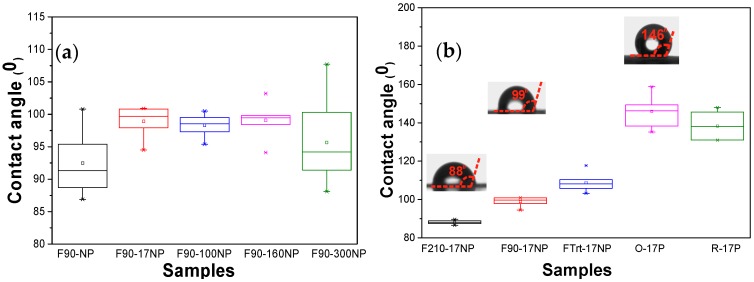
Contact angle of the SiNPs/PVDF nanocomposites: (**a**) PVDF with the SiNPs with different diameters processed at 90 °C and (**b**) SiNPs/PVDF samples with silica nanoparticles (17 nm) with different morphologies.

Material surface characteristics are essential in determining cell response in tissue engineering applications. For this reason, the static CA was measured on the different SiNPs/PVDF nanocomposites and the values are presented in Figure 5.

The introduction of the Si nanoparticles increases the CA values, independently of the diameter of the silica nanoparticles [18], to around 100° excepting for the samples with silica nanoparticles with the highest diameter (F90-300NP). This increase is attributed to the hydrophobic properties of the silica nanoparticles [18]. Samples with nanoparticles with the highest diameter show a higher range of CA values, which is explained by the variation in the diameter of the nanoparticles, as observed in Figure 1. Regarding Figure 5b, the CA for the composite samples with the smallest silica nanoparticles show that the CA of PVDF fibres increases significantly compared to the one of PVDF films, and the CA of the oriented PVDF fibres is slightly higher than the one for randomly oriented PVDF fibres, showing a contact angle of 146.0 ± 7.2°. These results support the idea that the increase in the hydrophobicity of electrospun samples is mainly related to the membrane morphology [8], with the fibres being significantly more hydrophobic than films. In the case of PVDF films, the CA is also higher for films with higher porosity, as already reported for pristine films [43].

#### 3.2.4. Structural Properties and Electroactive Phase Content of the Nanocomposites

**Figure 6:**
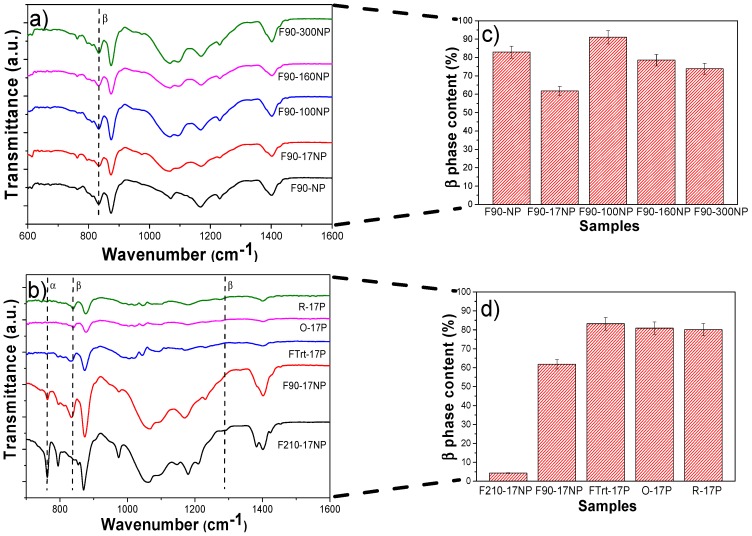
FTIR spectra of (**a**) neat PVDF and SiNPs/PVDF nanocomposites with silica nanoparticles of different diameters processed at 90 °C and (**b**) different morphologies of SiNPs nanocomposites prepared with the smallest nanoparticles. The β-phase content for the different sample is represented in (**c**,**d**).

FTIR-ATR spectra allow us to identify and quantify (Equation (2)) the polymer phase present in the samples and, therefore, to evaluate possible modifications induced by the introduction of silica nanoparticles (Figure 6).

Figure 6a shows the FTIR spectra of the different samples prepared at 90 °C as well as the corresponding quantification of the β-phase content (Figure 6c, calculated after Equation (1)). The characteristic bands of β PVDF (840 cm^−1^) is present in all samples, with low traces of α-PVDF (bands at 766, 855 cm^−1^), with the exception of F210-17NP. This is mainly attributed to the processing temperature [47], which mainly governs the solvent evaporation kinetics and the polymer crystallization in the β phase for processing at temperatures below 90 °C [44]. The introduction of SiNPs in PVDF does not significantly change the β-phase content, independently of the SiNPs content and average diameter. The β-phase value of pristine PVDF is 83 ± 3.3% and for the nanocomposites F90-17NP, F90-100NP, F90-160NP and F90-300NP, is 62 ± 2.5, 91 ± 3.6, 79 ± 3.1 and 74 ± 3, respectively (Figure 6c). On the other hand, Figure 6d shows that, depending on the nanocomposites’ morphology, the polymer crystallizes in different phases, mainly due to the different processing conditions. Thus, electrospinning involves room-temperature solvent evaporation and polymer stretching during jet formation, both favourable conditions for the crystallization of the polymer fibres in the β phase [49]. With respect to the films, the F210-17NP nanocomposite, which is processed by a melting and recrystallization process, crystallizes in the α-phase and shows that the addition of SiNPs does not induce the nucleation of the electroactive β-phase of the polymer, as observed in previous study with Fe_3_O_4_ spherical nanoparticles [51]. On the other hand, the porous samples, as well as the fibres, are prepared after solvent evaporation at room temperature, conditions leading to the crystallization in the β-phase. This fact is not affected by the introduction of the nanoparticles. Thus, it is concluded that the presence of the nanoparticles does not induce strong interactions with the polymer chain, leading to the nucleation of a specific phase, as observed with other fillers such as CoFe_2_O_4_ [52] and NaY zeolite [43]. Thus, processing temperature and solvent/polymer ratio remain the main factors determining polymer phase content in those composites [39,44].

#### 3.2.5. Thermal Behaviour of the Nanocomposites

**Figure 7:**
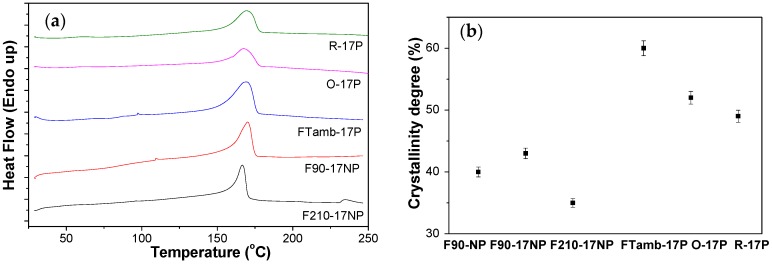
(**a**) DSC thermographs and (**b**) degree of crystallinity of the SiNPs/PVDF nanocomposites with different morphologies and with the fillers of lowest average diameter.

The DSC scans allow us to determine the melting temperature and the degree of polymer crystallinity (Figure 7).

All the samples show an endothermic peak around 168 °C corresponding to the polymer melting of the crystalline phase [44], thus, processing conditions and the incorporation of the filler do not affect the melting temperature. The degree of crystallinity was calculated (Equation (2)) from the enthalpy of the melting peak of the DSC thermograms. It was noticed that the samples prepared by solvent evaporation at 90 °C and after melting and recrystallization showed a lower degree of crystallinity than the samples prepared by solvent evaporation at room temperature, which also includes electrospun samples (Figure 7b). The pristine PVDF film processed at 90 °C shows a degree of crystallinity of ≈40%, which slightly increases with the introduction of the SiNPs and with the size of the SiNPs, being 43% for F90-17NP and 55% for F90-160NP (data not shown). Relative to the different morphologies (Figure 7a), the endothermic peak value is lower for the sample processed at 210 °C, indicating a lower degree of crystallinity if the sample, attributed to the fillers acting as defects during the crystallization from the melt [53]. Inclusion of the nanoparticles in the fibres does not significantly alters the crystallinity degree of the O-17P (52%) and R 17P (49%) with respect to the pristine polymer-oriented fibres (50% [8]).

The latter is ascribed to the combined effect of solvent evaporation at room temperature and stretching during the crystallization process that overcomes the effect of the presence of NP.

#### 3.2.6. Mechanical Properties of the Nanocomposites

**Figure 8:**
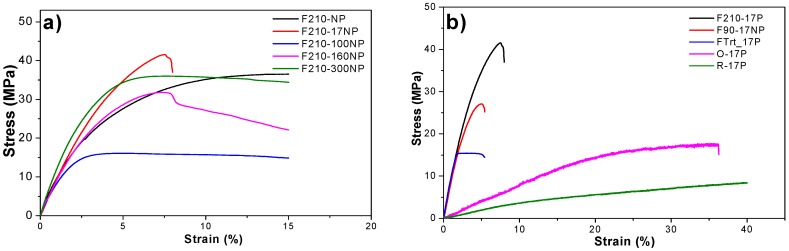
Stress‒strain curves for (**a**) SiNPs/PVDF nanocomposites with different SiNPs average diameters within the PVDF matrix and (**b**) for nanocomposites obtained after different processing conditions.

The mechanical properties of the materials are essential parameters to design a scaffold suitable for tissues with different mechanical characteristics. The characteristic mechanical strain-stress curves of samples with different morphology, filler type and content are presented in Figure 8.

Figure 8a shows the stress‒strain curves for the nanocomposites prepared with fillers with different average diameter after a melting process and Figure 8b refers to the nanocomposites with the same SiNPs (17 nm) after different processing conditions. Independently of the filler average diameter or processing conditions all samples show the typical mechanical behaviour of PVDF [54] characterized by the elastic region, yielding and plastic region, i.e., the typical behaviour of a thermoplastic elastomer.

**Figure 9:**
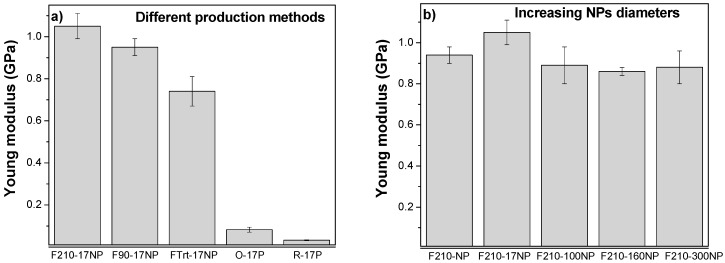
Young’s modulus of the SiNPs/PVDF nanocomposites varying (**a**) the processing method and (**b**) the average diameters of the SiNPs. The values are shown as mean ± SD.

The Young’s modulus of the samples was calculated from the linear zone of elasticity between 0 and 1% strain, as presented in Figure 9.

The characteristic features of the strain-stress curves are similar for all the materials, demonstrating that the mechanical characteristics are not strongly dependent on nanoparticle diameter. Furthermore, the introduction of particles with different diameters does not significantly affect the Young’s modulus of the pristine PVDF (F210-NP) −0.94 ± 0.04 GPa. However, a slight improvement in the Young’s modulus is observed for samples prepared with smaller silica nanoparticles (F210-17NP): 1.05 ± 0.06 GPa; this is in line with reports showing that the modulus increases as the particle size decreases [55]. Relative to the different production methods for the polymer films, F210-NP, F90-17NP and Frt-17P, it is observed that the more porous the structure, the lower the Young’s modulus, 0.83 ± 0.16 GPa for FTrt-17P. On the other hand, oriented fibres (O-17P) show a higher Young’s modulus (0.082 ± 0.012 GPa) than the random fibre samples (R-17P) (0.032 ± 0.002 GPa) due to the larger number of fibres along the stretch direction [8].

Relative to the other samples, the production method has a relevant influence on their mechanical response, as the samples prepared at room temperature by solvent evaporation showed a lower Young’s modulus than those obtained at 210 °C due to the porous nature of the former and the compact structure of the latter, as was also visible in the SEM images (Figure 3).

### 3.3. Cell Culture Studies

**Figure 10:**
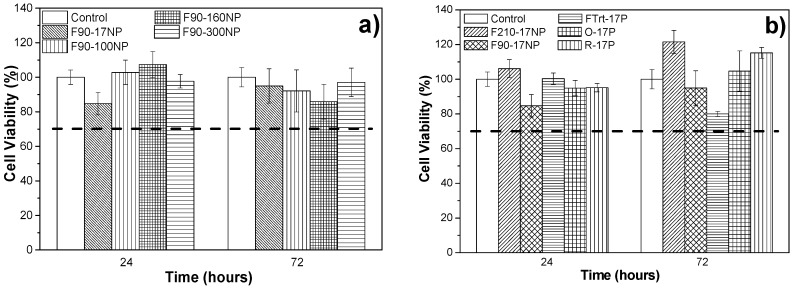
Cytotoxicity indirect test of (**a**) samples prepared with nanoparticles of different diameters and prepared by solvent evaporation at 90 °C and (**b**) samples prepared with SiNPs of 17 nm diameter after different processing methods and therefore with different morphologies.

In order to explore the potential use of the developed materials in tissue engineering applications, it is necessary to evaluate the putative cytotoxicity of the samples. The study of metabolic activity of C2C12 myoblasts, evaluated with the MTS assay, was applied to all samples and the results for 24 and 72 h are presented in Figure 10. Thus, the effects associated with introducing a fluorescent SiNP with different sizes are analysed, as well as the effect of the different microstructures/morphologies.

It has already been reported that PVDF is biocompatible and shows no cytotoxicity to C2C12 cells for 24 or 72 h [29,38]. The SiNPs are also biocompatible for many cells including C2C12 myoblasts [56,57,58]. It is important to notice that in the polymer composites silica nanoparticles are within the polymer films, avoiding any possible cytotoxic effects from the particles themselves. This is confirmed by the results of the cytotoxic assays of Figure 10. Once PVDF is a non-biodegradable polymer, there is also no risk of the particles leaching out from the films.

Thus, Figure 10 shows that none of the samples are cytotoxic, independently of the nanoparticle diameter and of the material morphology. It is important to note that, despite both materials being biocompatible, the result is not evident, as polymer‒filler interface effects or solvent retained in the nanoparticles or in the interface areas, can lead to cytotoxic effects. According to the ISO standard 10993-5, samples are considered cytotoxic when cells suffer a viability reduction larger than 30%. The measured cell viability values are all higher than 70%, confirming the cytocompatibility of the SiNPs/PVDF nanocomposites.

C2C12 myoblasts were used in previous studies to analyse cell proliferation of cultures grown on porous [59] and non-porous [38] PVDF films as well as fibres [38], with the verification that C2C12 cells proliferate better on piezoelectric β-PVDF “poled” samples. The samples obtained in this work were studied to determine the suitability for tissue engineering applications, namely muscle tissue.

**Figure 11:**
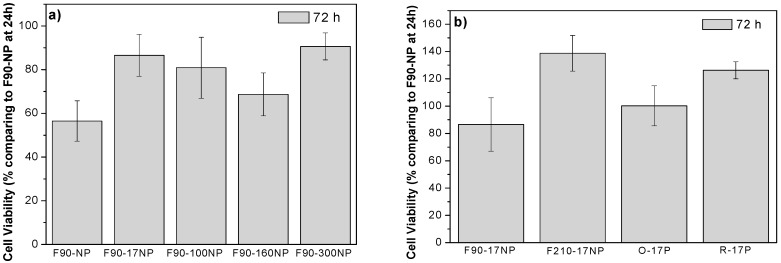
Cell proliferation of C2C12 cells seeded on (**a**) SiNPs/PVDF samples prepared at 90 °C with different sized nanoparticles and (**b**) SiNPs/PVDF samples with different morphologies.

**Figure 12:**
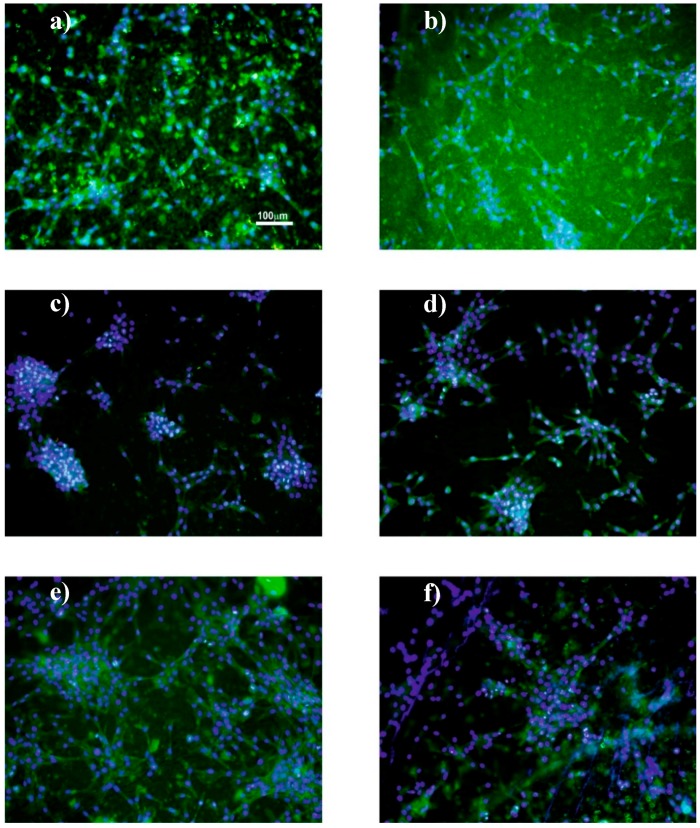
Representative images of C2C12 myoblast culture after 72 h on (**a**) F90-17NP, (**b**) F90-100NP, (**c**) F90-160NP, (**d**) F90-300NP, (**e**) F210-17NP and (**f**) FTrt-17P samples (nucleus stained with DAPI—blue and cytoskeleton stained with FITC—green). Scale bar = 100 µm for all the samples.

**Figure 13:**
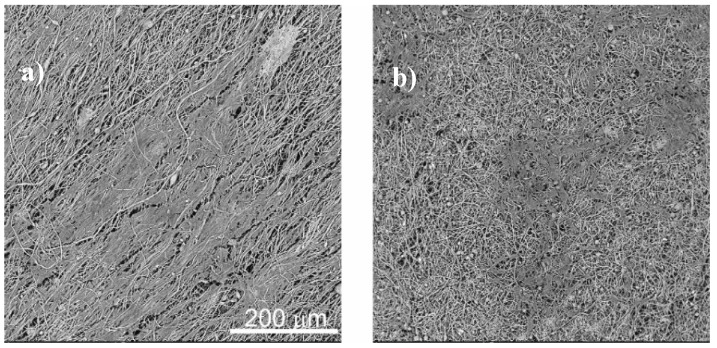
Cell morphology obtained by SEM of C2C12 myoblasts seeded on PVDF fibres: (**a**) O-17P and (**b**) R-17P, after three days of culture. The scale bar is 200 µm for all samples.

MTS (Figure 11), immunofluorescence (Figure 12) and SEM (Figure 13) assays were used to assess cell viability and morphology in the different samples. Relative to the proliferation results (Figure 11), the cell viability has been obtained in relation to the sample of F90-NP at 24 h.

(3) $$\text{Cell Viability}\left(\%\right)=\left(\frac{\text{Absorbance of samples at 72 h}}{\text{Absorbance of F90-NP at 24 h}}\times 100\right)-\text{cell viability of F90-NP at 24 h}$$

Figure 10 shows that the cell viability of the samples increases after 72 h of cell culture, independently of the SiNPs’ diameters (Figure 11a) and the morphology of the materials (Figure 11b), when compared with the sample without particles (F90-NP). No significant differences are observed between the samples and the negative control (F90-NP), revealing that C2C12 myoblast proliferation is not affected by the presence of SiNPs in the PVDF matrix. In fact, it has been reported that SiNPs being included in different polymers improves the cell attachment and proliferation, and enhances cellular processes [60,61], which is in agreement with the obtained results.

Cell cytoskeleton morphology, viability and adhesion were analysed by fluorescence microscopy for porous and non-porous films and SEM for fibre samples.

Independently of the nanoparticles’ diameters and the sample morphology, it is observed that the cell behaviour is similar. Bigger cell agglomerates (and larger nanoparticle agglomerates) are observed with the increasing nanoparticle diameter of the samples (Figure 12a–d). This fact is associated with the interaction between serum proteins and nanoparticles present on the PVDF matrix, as it has been reported that a negative surface charge enhances the adsorption of proteins with isoelectric point more than 5.5 such as immunoglobulin G (IgG) that can be important for C2C12 myoblasts [62,63]. Cell cultures on PVDF fibres prepared with the smaller silica nanoparticles were analysed by SEM and Figure 13 shows the cell morphology of C2C12 cells after 72 h of cell culture on oriented and random PVDF fibre nanocomposites.

These representative images demonstrate that, in the presence of a fibrillar microstructure, the muscle cells orientate their cytoskeleton along the fibres, which is in agreement with the literature [38]. In this way, in the presence of oriented fibres, the cells share a similar architecture to the natural muscle cells in living systems.

Thus, the overall results prove the potential of the use of SiNPs/PVDF piezoelectric nanocomposites for muscle tissue engineering. Physical and chemical stimuli are important factors to obtain tissues with characteristics similar to those of natural living tissues in the human body, developing therefore specific biomimetic microenvironments for different tissues according to their specific biophysico-chemical needs. The developed platform presents nanocomposites with different morphologies (membranes and fibres), piezoelectric β phase and SiNPs diameter (from 17 to 300 nm), which makes it an interesting and complete platform for tissue engineering.

Furthermore, this platform will allow further studies applying mechanical stimuli on the nanocomposites obtained in this work with specific bioreactors [36] applying mechanical and/or mechanoelectrical stimuli. It may also take advantage of the SiNPs’ capacity to include specific biomolecules or to develop drug delivery systems, or, more specifically, differentiation factors to promote directed myogenic differentiation. This will not only allow a deeper knowledge of the stimuli necessary for muscle tissue regeneration, but also lead to more effective therapies.

## 4. Conclusions

Different parameters important for tissue engineering, such as materials morphology, porosity and the PVDF electroactive phase, are modified in the obtained membranes.

Different diameters of silica nanoparticles have been introduced within the PVDF polymer matrix to obtain multifunctional samples for tissue engineering applications.

It is observed that the introduction of the SiNPs fillers in the PVDF matrix decreases its wettability. Furthermore, it is shown that the filler diameter does not significantly affect the properties of the polymer matrix, such as physico-chemical, thermal and mechanical properties.

Cytotoxicity assays with C2C12 cells show no cytotoxicity associated with neat PVDF and composites with different SiNPs diameters and sample morphologies.

Thus, it is demonstrated that the developed platform of PVDF materials with silica nanoparticles demonstrates potential for tissue engineering applications, allowing us to develop electromechanically active microenvironments with different morphologies with SiNPs, allowing protein functionalization and/or controlled release of specific drugs and/or growth or differentiation factors according to the targeted application.

## Figures and Tables

**Table 1:** Experimental details used for the preparation of the SiNPs.

Particle Diameter (nm)	EtOH (g)	H_2_O (g)	PDI Solution (mL)	NH_3_ (mL)	TEOS (mL)	Reaction Temperature (°C)
17	84.13	7.99	3	1.51	4.46	50
100	105.73	4.65	4	6.68	9.00	30
160	53.18	11.03	4	2.67	4.46	50
300	53.18	11.03	4	2.67	4.46	30

**Table 2:** Denomination, relevant preparation conditions and morphology of the PVDF and nanocomposite samples prepared in this work.

Morphology	Temperature (°C)	Time to Melt/Dry	Diameter of the Nanoparticles	Samples Morphology (P: Porous; NP: Non-Porous)	Denomination
**Films (F)**	90	30	---	NP	F90-NP
			17	NP	F90-17NP
			100	NP	F90-100NP
			160	NP	F90-160NP
			300	NP	F90-300NP
	210	10	17	NP	F210-17NP
	Room temperature (Trt)	----		P	FTrt-17P
**Oriented fibres (O)**	----	----		P	O-17P
**Random fibres (R)**	----	----		P	R-17P
